# Discovery of Phototoxic Metal Complexes with Antibacterial Properties via a Combinatorial Approach

**DOI:** 10.1021/acs.inorgchem.4c05414

**Published:** 2025-02-28

**Authors:** Timothy Kench, Nasima Sultana Chowdhury, Khondaker Miraz Rahman, Ramon Vilar

**Affiliations:** † Department of Chemistry, Imperial College London, White City Campus, 82 Wood Lane, London W12 OBZ, U.K.; ‡ Institute of Pharmaceutical Science, King’s College London, Franklin-Wilkins Building, 150 Stamford Street, London SE1 9NH, U.K.

## Abstract

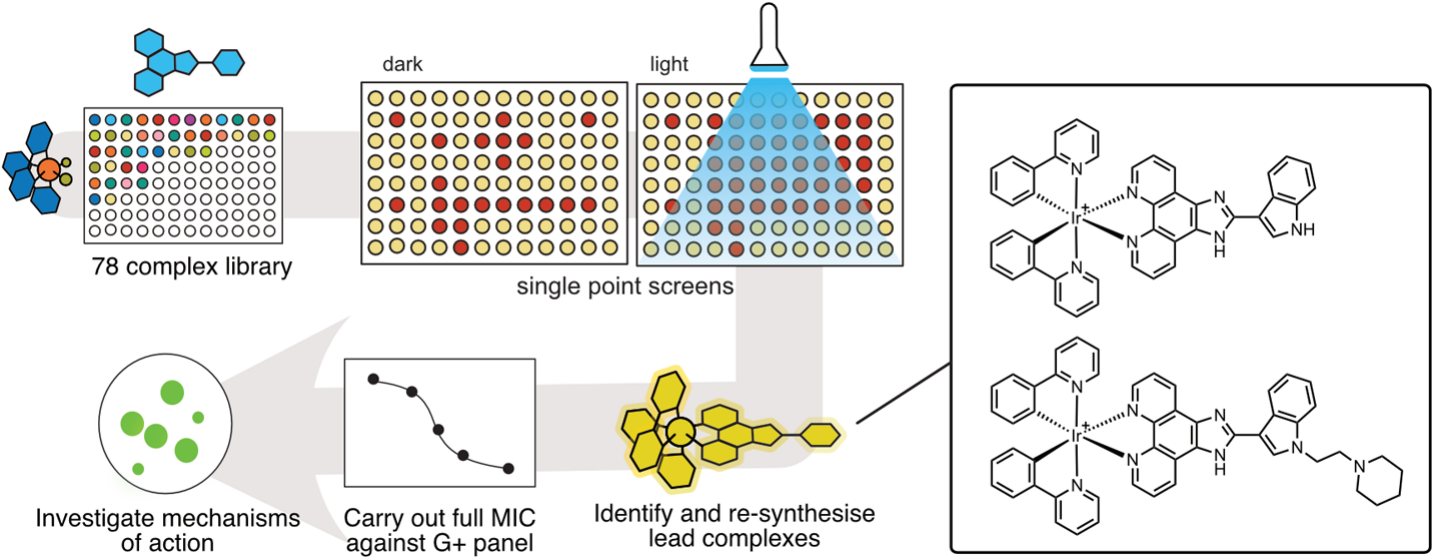

Antimicrobial resistance is one of the biggest global healthcare challenges. Therefore, there is an urgent need to develop new molecules that display distinct antibacterial properties to overcome resistance. With this aim, we have developed a combinatorial and semiautomated platform to synthesize and screen a library of 78 compounds against Gram-positive and Gram-negative bacteria. This library is based on octahedral iridium(III) complexes with general formula [Ir(**CN**)_2_(**NN**)]Cl (where **CN** are cyclometallating polyaromatic ligands and **NN** are phenanthroline-imidazole or dipyridophenazine derivatives) which are known to generate reactive oxygen species (ROS) upon light irradiation. From the initial screen of the entire library (in the dark and under light irradiation) against *Escherichia coli* and *Staphylococcus aureus*, we show that this scaffold is highly effective at inhibiting growth of Gram-positive bacteria at an intermediate dose (16 μg/mL), displaying a hit rate of >30% in the dark and rising to 56% under light irradiation. Six complexes were selected for further studies against a panel of five Gram-positive strains, allowing us to identify two lead complexes with MICs as low as 2 μg/mL. These complexes were studied in more detail to establish their mode of action using a time-kill study against the *S. aureus* USA300 strain.

## Abstract 2

A combinatorial and semiautomated approach has been used to synthesize and screen a library of 78 photoactive iridium(III) complexes against Gram-positive and Gram-negative bacteria. Through three subsequent rounds of testing, two lead compounds were identified which display high activity upon light irradiation against *S. aureus* US300.

## Introduction

Antimicrobial resistance (AMR) is a critical public health issue, directly causing over 1.27 million global deaths in 2019 with this number predicted to reach 10 million by 2050.^1^ In addition to its devastating impact on health, AMR also has significant economic consequences, with the World Bank estimating that by 2050 AMR could result in US$ 1 trillion additional healthcare costs.^2^ The main causes of AMR are associated with the misuse and overuse of antibiotics not only in humans but also in animals and plants. On the other hand, the arsenal of effective antibiotics to resistant bacteria is rapidly decreasing. Therefore, there is an urgent need to develop new molecules with distinct and varied modes of action for the treatment of bacterial infections resistant to current antibiotics.^3,4^

There are three main mechanisms by which bacteria develop antibiotic resistance: (i) enzymatic degradation of drugs; (ii) reduced drug uptake (either by decreased membrane permeability or increased efflux pump activity); and (iii) modifications of bacterial biomolecules (mainly proteins) that are drug targets.^5,6^ In addition to these three main mechanisms, the formation of bacterial biofilms has also been shown to drive antibiotic resistance. Thus, the discovery of new drugs often focuses in overcoming these mechanisms of resistance. In addition, the development of new antibiotics needs to consider the activity of the drugs not only against the target bacteria but also the host organism to prevent unwanted toxicity against the host.

To address some of these challenges, metal complexes are being studied as potential antibiotics since their varied geometries can provide a very broad and unexplored chemical space not easily achievable with purely organic molecules.^7^ Furthermore, metal centers can themselves be active (e.g., copper and silver have been known for a long time to have antibacterial properties^8^) and/or provide unique functionalities to the resulting metal complexes (e.g., photochemical, catalytic or redox properties). Evidence for the great potential of metal complexes as antibacterial agents comes from an analysis of compounds screened by the Community for Open Antimicrobial Drug Discovery (CO-ADD).^9^ Up to July 2019, over 900 metal complexes had been reported in this database displaying a 9.9% hit rate as compared to purely organic compounds (with a hit rate of 0.87%). A wide range of complexes containing different metals (including Mn, Co, Zn, Ru, Ag, Eu, Ir, and Pt) showed to be active against Gram-positive and/or Gram-negative bacteria. Some of the best performing metal-containing compounds displayed activities down to the nanomolar range against several pathogens.

Despite the growing number of reports showing the potential of metal complexes as antibacterial agents, the discovery of active compounds is challenging, lengthy, and not very efficient. Furthermore, there is also a lack of consistent and systematic structure activity relationship (SAR) studies for metal complexes that display antibacterial properties. To address these challenges, the development and screening of combinatorial libraries of metal complexes have been recently reported. As discussed in further detail below, in the context of antimicrobial screening, this has included ruthenium–arene Schiff base complexes^10−12^ and manganese(I) tricarbonyl complexes.^13^ However, both of these families of complexes contain labile ligands and can be prone to speciation^14^ (or indeed, rely on it for activation), which can be a limitation in metallodrug discovery.^15^ An alternative approach is to rely on more substitutionally inert metal complexes, such as those containing ruthenium(II) or iridium(III). Indeed, this chemistry is also amenable to combinatorial synthesis, with iridium(III)^16^ and rhodium(III)^17^ polypyridyl libraries having been prepared for photocatalytic applications and ruthenium(II) and iridium(III) polypyridyl complex libraries having been tested for potential anticancer properties.^18,19^

The photochemical properties of second and third row transition metal complexes (including ruthenium(II), osmium(II), iridium(III), and platinum(II) among others), offer additional advantages, such as the potential to use photodynamic therapy (PDT) as treatment.^20,21^ In PDT, reactive oxygen species (ROS) are generated upon light irradiation of a suitable photosensitizer. Since ROS are generated only in areas where the photosensitizer is accumulated and irradiated, this process allows to damage diseased tissue (e.g., cancerous tissue) selectively.^22−24^ PDT also offers several notable benefits in the field of antifungal and antimicrobials (termed antimicrobial photodynamic therapy, aPDT); since ROS have no specific cellular target, it makes developing mechanisms of resistance for bacteria much harder. Bacterial biofilms, which are able to dramatically increase antimicrobial resistance, can also be destroyed by ROS.^8,25^ In addition, the high degree of spatiotemporal control means that it could be used to selectively treat wounds or disinfect medical device surfaces without the need for broad-spectrum antibiotics.^26^ Several iridium complexes have been studied as antimicrobial PDT agents including cyclometalated iridium(III) complexes coordinated to ligands, such as dipyrrinato,^27^ 2-phenylbenzimidazole derivatives,^28^ and a range of substituted phenanthrolines,^29^ among others.^30,31^

**Figure 1:**
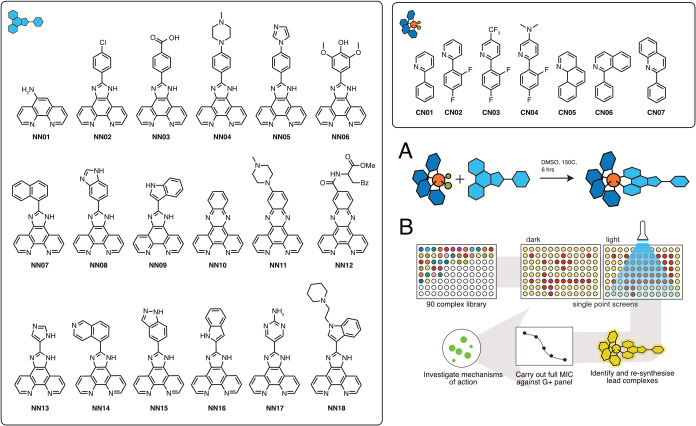
Project workflow: (A) High-throughput synthesis of previously reported library and (B) workflow to identify phototoxic iridium(III) complexes for antibacterial applications.

We recently reported an automated and combinatorial workflow for the synthesis, characterization, and cellular screening of iridium(III) complexes with general formular [Ir(**CN**)_2_(**NN**)]Cl (see Figure 1).^19^ The rationale for choosing this type of complex is based on their known ability to generate ROS upon visible light irradiation. In our previous study, we reported the detailed protocol to generate the library of complexes shown in Figure 1. For this, a set of iridium(III) dimers coordinated to cyclometallating ligands **CN01**-**07** and a set of **NN01-18** ligands were prepared. The iridium(III) dimers were cleaved in dimethyl sulfoxide (DMSO) to form complexes with the general formula [Ir(**CN**)_2_Cl(DMSO)] (where **CN** = **CN01**-**CN07**), which subsequently reacted cleanly with **NN** ligands to generate the final combinatorial library of 78 [Ir(**CN**)_2_(**NN**)]Cl complexes.^19^ We also demonstrated in our previous study that these complexes could be taken forward without the need for further purification, testing their photophysical properties, ability to generate ROS, and their toxicity against cancer cell lines in the dark and after visible light irradiation. Through this process, we were able to identify specific compounds which were effective at generating ROS and had low cytotoxicity in the dark against mammalian cancer cells but were highly phototoxic after light irradiation. Importantly, we also observed a wide range of behavior across the library, with some complexes seemingly being very good at generating reactive oxygen species but not effectively taken up by cells.

**Figure 2:**
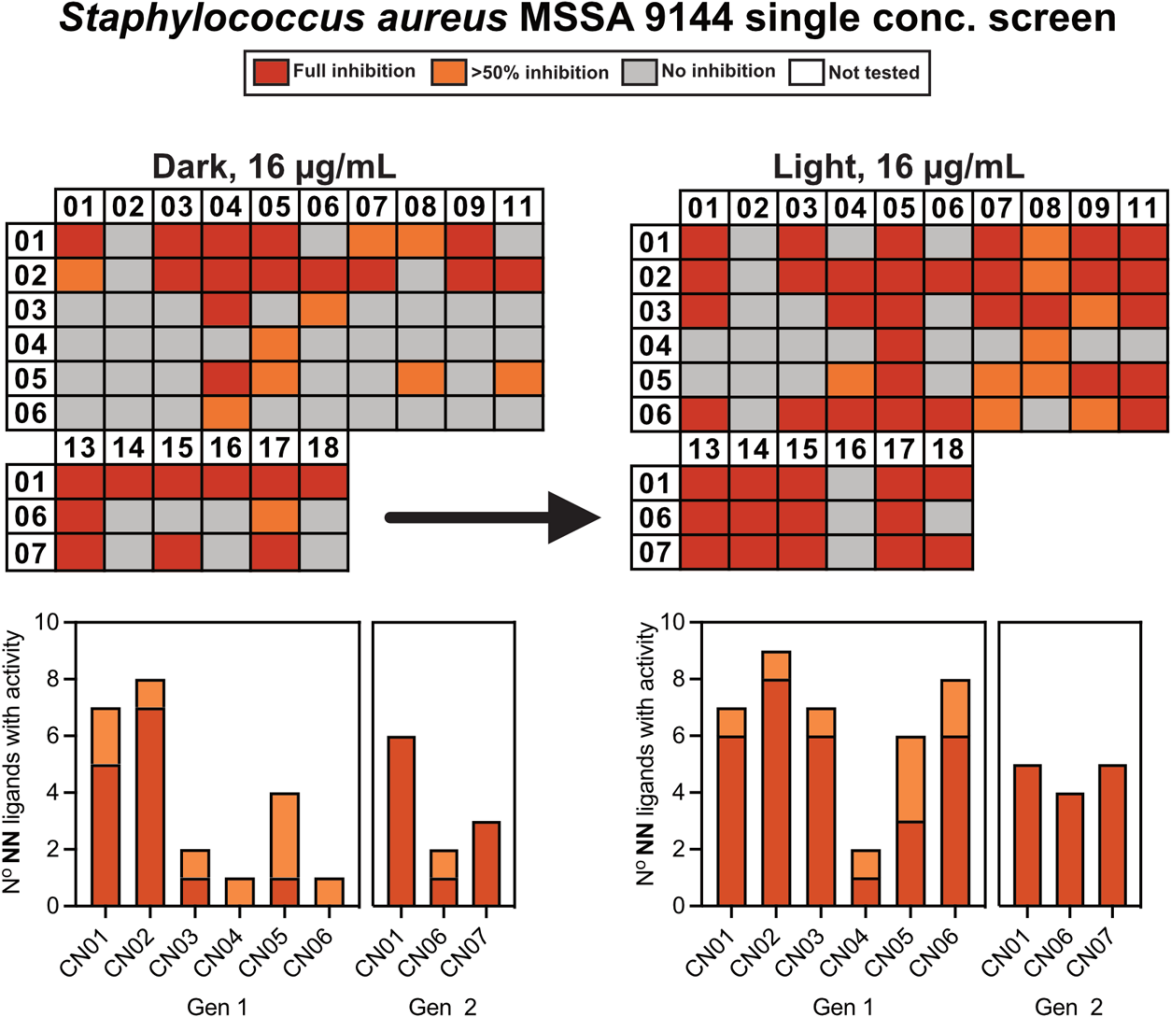
Single-point screening workflow. Complexes were screened at a single concentration first in the dark, followed by an experiment in which 96-well plates were irradiated with low-intensity (1.2 J/s) blue light for 12 min.

**Figure 3:**
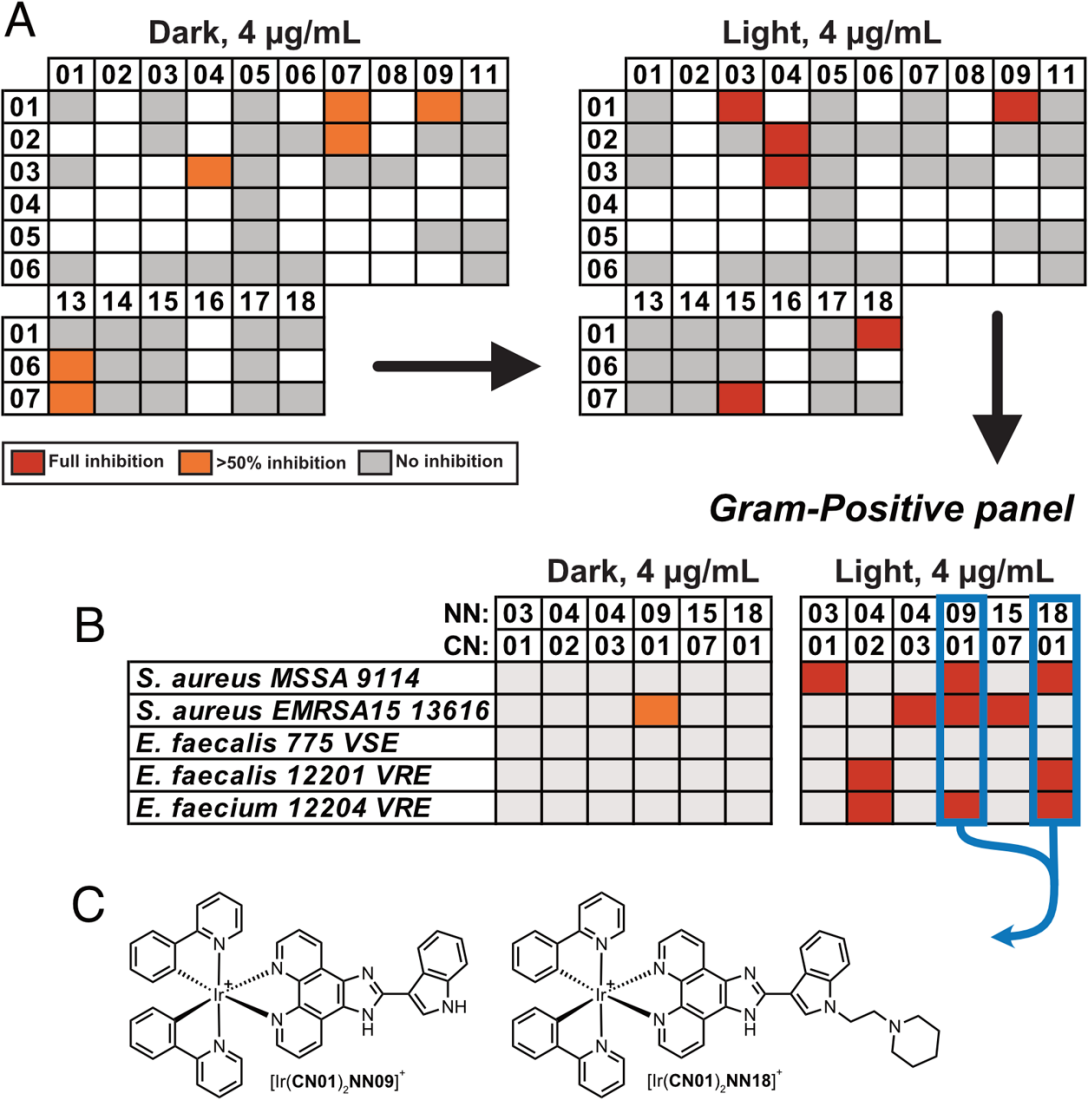
Next screening stages: (A) screening concentration dropped to 4 μg/mL for both light and dark conditions; (B) six lead complexes were taken forward and screened against a panel of five Gram-positive strains. From these results, the two complexes showing the broadest spectrum of activity were identified and resynthesized; (C) the chemical structures of the two lead complexes.

Given the differing mechanisms of uptake for mammalian and bacterial cells, and considering previous studies showing the antimicrobial properties of various iridium(III) complexes,^27−32^ herein, we report studies to determine the potential of the compounds in this library to act as antibacterials. By screening the 78 complexes which had also been tested against mammalian cells, we aimed to identify certain structural motifs which might be particularly selective for one or the other. We report a workflow to effectively screen a large number of iridium(III) complexes for their antibacterial properties; in this workflow, a series of single-point screens were used to identify a handful of lead compounds, which were then resynthesized via conventional means and their antibacterial properties investigated in further detail (Figures 2 and 3).

## Experimental Section

### High-Throughput Library Synthesis

Synthetic detail of how the library of 78 iridium(III) complexes was generated, can be found in our previous publication.^19^

### Preparative Synthesis

^1^H and ^13^C NMR spectra were recorded on a Bruker Avance 400 MHz (with a Bruker SampleJet attachment for the calibration and final complex syntheses). LCMS analysis was carried out on an Agilent 1260 Infinity with a Raptor C18 column (50 mm × 2.1 mm, 2.7 μm particle size). A 2 min gradient from 5 to 95% MeCN in water was used and supplemented with 0.1% formic acid. All chemicals were purchased from Sigma-Aldrich, Fluorochem, or VWR and used without further purification. Complex [Ir(**CN01**)(μ-Cl)]_2_ was prepared as previously reported.^33^

### [Ir(CN01)_2_(NN09)]

[Ir(**CN01**)(μ-Cl)]_2_ (10.7 mg, 0.02 mmol) and **NN09** (6.7 mg, 0.02 mmol) were combined in a mixture of chloroform (15 mL) and methanol (5 mL) and stirred at 40 °C overnight. Next, the solvent was removed under reduced pressure and the compounds were purified by semipreparative high-performance liquid chromatography (HPLC) (5–95% MeCN in H_2_O, 0.1% formic acid). ^1^H NMR (400 MHz, DMSO) δ 11.78 (s, 1H), 9.35–9.28 (m, 2H), 8.76 (dd, *J* = 6.4, 3.1 Hz, 1H), 8.54 (s, 2H), 8.28 (d, *J* = 8.3 Hz, 2H), 8.08 (d, *J* = 4.9 Hz, 2H), 8.05–7.93 (m, 4H), 7.93–7.84 (m, 2H), 7.52 (dd, *J* = 6.0, 1.6 Hz, 3H), 7.24 (dt, *J* = 6.0, 3.5 Hz, 2H), 7.12–6.92 (m, 6H), 6.32 (dd, *J* = 7.5, 1.2 Hz, 2H). Electrospray ionization-mass spectrometry (ESI-MS): calculated for [C_43_H_29_IrN_7_]^+^ 836.2114; found 836.2126.

### [Ir(CN01)_2_(NN18)]

[Ir**(CN01**)(μ-Cl)]_2_ (10.7 mg, 0.02 mmol) and **NN18** (8.9 mg, 0.02 mmol) were combined in a mixture of chloroform (15 mL) and methanol (5 mL) and stirred at 40 °C overnight. Next, the solvent was removed under reduced pressure and the compounds were purified by semipreparative HPLC (5–95% MeCN in H_2_O, 0.1% formic acid). ^1^H NMR (400 MHz, DMSO) δ 9.28 (d, *J* = 8.3 Hz, 2H), 8.77–8.70 (m, 1H), 8.55 (d, *J* = 4.4 Hz, 2H), 8.28 (d, *J* = 8.2 Hz, 2H), 8.09 (d, *J* = 4.9 Hz, 2H), 8.05–7.94 (m, 4H), 7.89 (td, *J* = 7.8, 1.5 Hz, 2H), 7.66–7.59 (m, 1H), 7.52 (d, *J* = 5.8 Hz, 2H), 7.28 (h, *J* = 6.4 Hz, 2H), 7.11–6.92 (m, 6H), 6.32 (d, *J* = 7.3 Hz, 2H), 4.41 (t, *J* = 6.7 Hz, 2H), 2.74 (t, *J* = 6.7 Hz, 2H), 2.44 (m, 4H), 1.48 (m, 4H), 1.37 (s, 2H). ESI-MS: calculated for [C_50_H_42_IrN_8_]^+^ 947.3162; found 947.3198.

### Antimicrobial Screening of Compounds Using Single Concentrations of 16 and 4 μg/mL

Overnight cultures of *Escherichia coli* 12923 and *Staphylococcus aureus* MSSA 9144 were prepared in Tryptic Soy Broth (Cat. No. 22092, Sigma-Aldrich) and grown overnight at 37 °C with shaking at 200 rpm. The compounds were diluted to concentrations of 16 and 4 μg/mL. A 100 μL aliquot of each dilution was added to two wells (treated and blank) in a 96-well flat-bottom plate (Cat. No. 655180, Greiner). Two sets of plates were prepared: one without irradiation and one with irradiation.

The overnight bacterial cultures were diluted 1:10 in phosphate-buffered saline (PBS), the optical density at 600 nm (OD_600_) was measured, and the cultures were further diluted to an OD_600_ of 0.01. A 100 μL aliquot of the diluted culture was added to the wells containing the compounds (except the blank wells) in a 1:1 ratio, yielding a starting bacterial density of ∼5 × 10^5^ CFU/mL. As the compounds were dissolved in DMSO, a 1% DMSO control (Cat. No. A13280.36, Thermo Scientific) was included. The plates were incubated at 37 °C. After 3 h, one set of plates was irradiated using an 8 mA light source for 12 min at a wavelength of 457 nm, then returned to the incubator, and further incubated for up to 20 h. The optical density at 600 nm was measured spectrophotometrically using a FLUOstar Omega Microplate Reader.

### Determination of Minimum Inhibitory Concentration (MIC) Using the Broth Microdilution Method

To determine MIC, overnight cultures of Gram-positive bacterial strains were prepared in Tryptic Soy Broth and grown overnight at 37 °C with shaking at 200 rpm. The compounds were serially diluted to concentrations ranging from 16 to 0.125 μg/mL in two sets of 96-well flat-bottom plates, with 100 μL of each dilution per well. The bacterial cultures were diluted to an OD_600_ of 0.01 and added to the serially diluted compounds in the plates (except the blank wells). A DMSO control was included for MIC detection. Plates were incubated at 37 °C for 3 h. One set of plates was irradiated and then returned to the incubator for up to 20 h. The MIC was determined as the lowest concentration of the compound with no visible growth (OD <0.1 after subtracting the blank OD). MIC50 was determined as the lowest concentration that resulted in a 50% reduction in bacterial growth compared to the positive control (bacteria without treatment).

### Determination of Minimum Bactericidal Concentration (MBC)

For MBC determination, 10 μL samples were taken from the wells showing no visible bacterial growth in the MIC assay. These samples were then spotted onto TSA agar plates under sterile conditions in a safety cabinet. The plates were incubated overnight at 37 °C and subsequently examined for bacterial regrowth. The lowest concentration of the compound that completely inhibited bacterial regrowth on the agar plate was recorded as the MBC.

### Cytotoxicity and phototoxicity

HEK293 cells were grown in high glucose Dulbecco’s modified Eagle medium (DMEM) containing 10% fetal bovine serum (FBS) at 37 °C with 5% CO_2_ in humidified air. For viability experiments, cells were seeded at a density of 10,000 cells per well in Greiner-Bio black μClear plates which had been coated in poly-d-lysine.

After 24 h, the corresponding iridium complexes were added to the cells at the appropriate concentrations and allowed to incubate for further 20 h. For the phototoxicity experiments, the plate was removed at the 3 h time point and irradiated for 12 min using a Lumidox 457 nm 96-LED array set to 8 mA (2 mW/cm^2^, a total of 1.2 J/s). After 20 h, the media was replaced with fresh media containing an MTS/PMS mixture as per the Promega protocol (MTS; 3-(4,5-dimethylthiazol-2-yl)-5-(3-carboxymethoxyphenyl)-2-(4-sulfophenyl)-2Htetrazolium, PMS; phenazine methosulfate). After an additional 4 h had passed, the absorbance at 490 nm (MTS) and 635 nm (background) was measured. Cell viability was calculated from the dose–response curve of absorbance (MTS – background).

### Time-Kill Assay

The time-kill assay determines bacteriostatic or bactericidal activity of compounds over time by exposing microorganisms to various concentrations (MIC and higher) of the test compounds. An overnight culture of *S. aureus* USA300 was diluted to an OD_600_ of 0.01 in vials designated for untreated (control) and treated samples. A 1:10 serial dilution of bacteria from the control vial was prepared in a 96-well plate (0 h time point), and 10 μL samples from each dilution were plated on agar. Test compounds were added to the treated vials at concentrations of 4 × MIC, and 8 × MIC. Vials were incubated at 37 °C with shaking.

Samples were collected at various time points (1, 2, 4, 6, 24, and 48 h), serially diluted, and plated on agar. For single irradiation, cultures in vials were transferred to a 96-well plate after 3 h, irradiated, transferred back to the vials, and returned to the shaking incubator. For double irradiation, the process was repeated at the 6 h time point. Agar plates were incubated overnight at 37 °C, and bacterial colonies were counted. Colony counts were expressed as CFU/mL and plotted against time as log CFU/mL. The antimicrobial activity of the compound was classified as bacteriostatic if the colony count showed a ≤ 3-log reduction in CFU/mL compared to the 0 h time point and classified as bactericidal if the reduction exceeded 3-log CFU/ml. Viable cells observed after the 24 h period were isolated, repassaged in the absence of the compound and reassayed, along with nontreated and nonirradiated *S. aureus* USA300, using the MIC determination protocol described above.

## Results and Discussion

As indicated above, we previously prepared a library of 90 iridium complexes over two generations.^19^ In the first, **NN01**-**12** was combined with **CN01**-**06** to form a 72-complex first-generation library. A subsequent second-generation of 18 complexes were then synthesized via the combination of **NN13**-**18** with **CN01**, **CN06**, and **CN07**, for a total of 90 complexes. From this library, for the studies herein presented, we excluded complexes with **NN10** due to their high toxicity against mammalian cells and **NN12** due to the presence of a persistent impurity seen across all complexes formed with this ligand. This led to a total 78 complexes taken forward for the screening presented in this work. The complexes were tested against representative Gram-negative (*E. coli* 12923) and Gram-positive (*S. aureus* MSSA 9144) strains at a single concentration of 16 μg/mL. The complexes were first tested in the dark, followed by an experiment in which the 96-well plate was irradiated using blue light (457 nm) for 12 min (total dose 1.2 J/s).

None of the iridium complexes showed any inhibition against *E. coli* in either the dark or when irradiated with blue light, while many complexes showed a measurable effect against *S. aureus* without and with light irradiation (see Figure 2). Of the 78 complexes, 24 showed full inhibition at 16 μg/mL without light irradiation, which represents a hit rate of over 30% (Table S1 and Figure S7). For comparison, a recently screened library of 288 ruthenium–arene complexes displayed a hit rate of 5.6% at a roughly equivalent concentration (20 μM) and a library of 420 manganese tricarbonyl complexes had a hit rate of 15% (although this library was screened at a lower concentration of 12.5 μM), emphasizing the potential of our iridium(III) scaffold to be used for antibacterial applications. Upon irradiation, a marked increase in activity was observed, with 44/78 complexes (56%) showing complete inhibition of bacterial growth with 9 (11%) showing >50% growth inhibition (Table S2 and Figure S8). Most of the nonactive complexes contain either **NN02**, **NN06**, **NN16** phenanthroline-imidazole ligands, or **CN04** as the cyclometallating ligand. Interestingly, **CN04** contains the same difluoro phenylpyridine scaffold as **CN02**, just with an additional dimethylamino functional group, which was thought could improve bacterial uptake/toxicity; however, complexes containing ligand **CN02** are the best performing compounds across both dark and light conditions. In line with their excellent ROS generating abilities,^19^ complexes containing **CN06** and **CN07** (and to lesser extent **CN05**) generally became much more toxic upon irradiation.

The selectivity of these complexes for *S. aureus* MSSA 9144 (Gram-positive) over *E. coli* 12923 (Gram-negative) bacteria is likely due to differences in their permeability. Gram-positive bacteria lack an outer membrane, making them more susceptible to interactions with large, hydrophobic metal complexes. On the other hand, hydrophilic compounds generally have a greater ability to cross the Gram-negative membrane and exhibit an overall better activity profile.^34,35^ Thus, the inactivity of these complexes against Gram-negative bacteria suggests that they are too hydrophobic, and specific hydrophilic modifications are necessary to widen their spectrum of activity.

Any complex showing full inhibition at 16 μg/mL was taken forward for the next stage, in which the screening concentration was dropped to 4 μg/mL for both light and dark conditions. Under these conditions, six complexes were partially active (>50% inhibition) in the dark, while a different six showed full growth inhibition after irradiation ([Ir(**CN01**)_2_(**NN03**)]^+^, [Ir(**CN02**)_2_(**NN04**)]^+^, [Ir(**CN03**)_2_(**NN04**)]^+^, [Ir(**CN01**)_2_(**NN09**)]^+^, [Ir(**CN07**)_2_(**NN15**)]^+^, and [Ir(**CN01**)_2_(**NN18**)]^+^), see Figure 3A. Again, an interesting comparison can be drawn between these antibacterial lead complexes and those identified as photoactive against HeLa and U2OS human cancer cells; the complexes most active against human cancer cells were dominated by those containing **CN06** and **CN07** ligands. Here, five of six lead complexes were with **CN01**-**3** and no complexes with **CN06** were deemed sufficiently toxic, indicating a preference for smaller, unsubstituted (**CN01**) or fluoro-containing (**CN02** and **CN03**) cyclometallating ligands.

These six complexes were then tested for their broad-spectrum properties against a total of five Gram-positive bacterial strains, including *Staphylococcus aureus* (MSSA 9144 and EMRSA15 13616, with the latter representing a methicillin-resistant strain), two *Enterococcus faecalis* strains (775 VSE and 12201 VRE, for vancomycin sensitive and resistant strains, respectively), and one *E. faecium* strain (12204 VRE, a generally more antibiotic-resistant strain than faecalis), see Figure 3B. Encouragingly, this class of iridium polypyridyl complexes appeared able to effectively kill antibiotic-resistant strains alongside their more sensitive counterparts. Indeed, while none of the complexes appeared active at 4 μg/mL against *E. faecalis* 775 VSE, this increased to two and three for *E. faecalis* 12201 VRE and *E. faecium* 12204 VRE, respectively.

From this panel, we selected two iridium complexes as our lead compounds, defined by those which showed the broadest activity, namely, [Ir(**CN01**)_2_(**NN18**)]^+^ and [Ir(**CN01**)_2_(**NN09**)]^+^, and full inhibition against three strains each (Figure 3B,C). These complexes were resynthesized in larger amounts and purified via conventional means (SI, Figures S1–S6). Interestingly, these complexes are structurally related, with both not only containing phenylpyridine (**CN01**) as the cyclometallating ligand but also an indole scaffold as the **NN** ligand: in the case of **NN09** the indolyl phenanthroline-imidazole derivative, whereas for **NN18** an ethylpiperidine derivative (Figure 3C).

**Figure 4:**
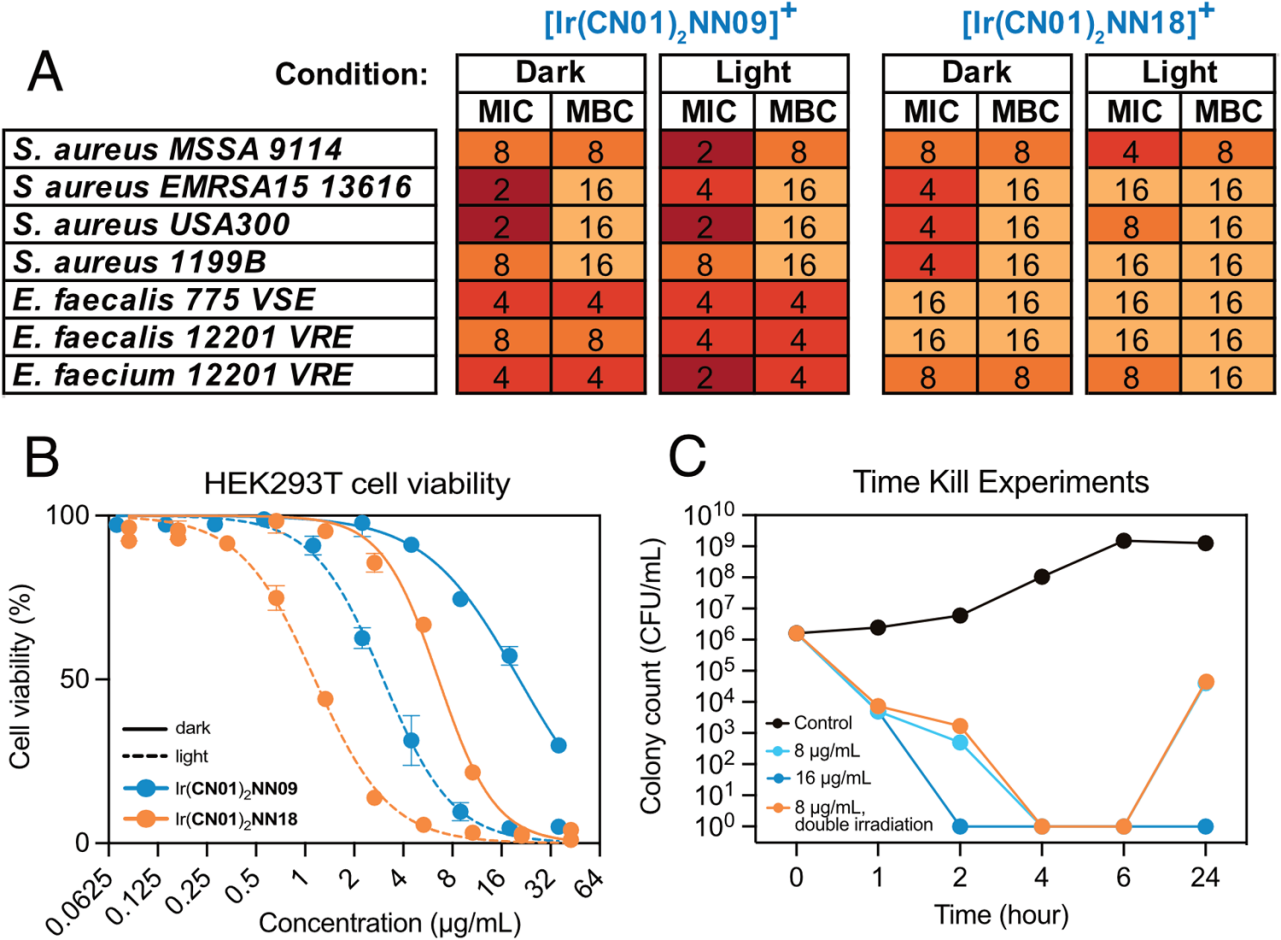
Lead compounds’ data: (A) minimum inhibitory concentration (MIC) and minimum bactericidal concentration (MBC) results for the two lead complexes against seven Gram-positive strains in the dark and after light irradiation (all units in μg/mL); (B) lead complexes tested against HEK293T cells for their light and dark toxicities. Cells were irradiated with the same dose of light as the bacterial experiments. Dark toxicity is indicated by a solid line, and light toxicity is indicated by a dashed line. (C) Time-kill assays with *S. aureus* USA300 at various time periods. A serial dilution was prepared from untreated *S. aureus* USA300 (Control) and plated out (time point 0). Different concentrations of [Ir(**CN01**)_2_(**NN09**)]^+^ were added to the rest of the vials containing *S. aureus* USA300 and incubated at 37°C. Samples were collected at different time points, prepared a serial dilution and plated out. [Ir(**CN01**)_2_(**NN09**)]^+^-treated bacteria were irradiated for 12 min using 8 mA light at 457 nm wavelength after 3 h of incubation. Bacterial colony was counted after overnight incubation at 37 °C. For the double irradiation dose, the cells were also irradiated at 6 h.

These complexes were then screened against a further extended panel of seven Gram-Positive strains, with the addition of *S. aureus* USA300 (a particularly resistant and clinically relevant strain of MRSA) and *S. aureus* 1199B (a multidrug resistant as opposed to methicillin-resistant strain). In this experiment, the minimum inhibitory concentration (MIC) was calculated for each complex against each strain under both light and dark conditions (Figure 4A). The calculated MICs showed that these two compounds to be highly active, as well as being in line with the results from the high-throughput screen. This also validated our experimental workflow, which involved initial screenings conducted without prior compound purification. Consistent with earlier findings, the complexes exhibited greater activity against methicillin-resistant strains compared to methicillin-sensitive strains. Of the two complexes, [Ir(**CN01**)_2_(**NN09**)]^+^ was not only more active in the dark (with MICs as low as 2 μg/mL in the case of the USA300 strain) but also showed a much more pronounced light-activated effect, with a 2–4 fold decrease in the MIC upon irradiation. This light-dependent activation offers an opportunity to treat localized infections, such as wound or surface infections, where localized light exposure is feasible. This strategy offers an opportunity for site-specific bacterial eradication without systemic toxicity. For [Ir(**CN01**)_2_(**NN18**)]^+^, the lowest MIC obtained was 4 μg/mL and only one of seven strains showed an increase in MIC upon light irradiation. This indicates that the two complexes might have different bacterial targets.

These complexes were also tested on the human hepatocyte cell lines HEK293T to assess their toxicity against healthy mammalian cells. For this experiment, the IC50s of the two lead complexes were determined both in the dark and under light irradiation with identical incubation times and light doses to those used in the antibacterial MIC experiments (Figure 4B). For [Ir(**CN01**)_2_(**NN09**)]^+^ and [Ir(**CN01**)_2_(**NN18**)]^+^, the dark IC50 values against HEK293T were 20.5 and 6.58 μg/mL, respectively (*cf*. MICs of 2–8 and 4–16 for the different Gram(+) strains investigated, Figure 3). As expected, upon irradiation, both [Ir(**CN01**)_2_(**NN09**)]^+^ and [Ir(**CN01**)_2_(**NN18**)]^+^ showed to be more toxic against HEK293T (IC50 of 3.04 and 1.15 μg/mL, respectively). This shows that for [Ir(**CN01**)_2_(**NN09**)]^+^, there is an up to 10-fold increase in toxicity against bacterial cells than healthy human cells in the dark. With light irradiation this selectivity does generally decrease, due the larger increase in toxicity seen in healthy cells. Unfortunately, for [Ir(**CN01**)_2_(**NN18**)]^+^ toxicity values were higher or around the same for healthy cells as for bacterial cells.

The ability of some compounds to show moderate to good activity against bacteria without light irradiation, while exhibiting enhanced activity after irradiation, can be beneficial for treating infection. Controlling activation to a level that is lethal to bacteria but nontoxic to human cells can reduce off-target effects. This allows for tailored or personalized treatment, depending on the causative bacteria; for some infections, activity in the dark mode may be sufficient, while for others, irradiation may be required.

Given the superior properties of [Ir(**CN01**)_2_(**NN09**)]^+^ and [Ir(**CN01**)_2_(**NN18**)]^+^, we performed further experiment to investigate whether these complexes were bacteriostatic or bactericidal. The minimum bactericidal concentration (MBC) values were determined following the MIC assay by plating samples from wells with no visible bacterial growth onto agar and incubating them overnight (Figure 4A). The results indicate variability in bactericidal activity across different strains. The MBC values for *S. aureus* MSSA 9144 and all three *Enterococcus* strains were found to be close to their MICs, suggesting that [Ir(**CN01**)_2_(**NN09**)]^+^ and [Ir(**CN01**)_2_(**NN18**)]^+^ have a strong bactericidal effect against these strains. In contrast, the higher MBC values observed for the MRSA strains *EMRSA*15 13616 and USA300 suggest that intrinsic resistance mechanisms in these strains may play a role in reducing the effectiveness of both iridium complexes in bacterial eradication. To further confirm the bactericidal mode of action for [Ir(**CN01**)_2_(**NN09**)]^+^, a series of time-kill assays were performed on*S. aureus* USA300 at 4× MIC (8 μg/mL) and 8× MIC (16 μg/mL). (Figure 4C). In these experiments, the bacteria were irradiated at the 3 h time point. The results showed that [Ir(**CN01**)_2_(**NN09**)]^+^ is bactericidal at 8× MIC (16 μg/mL), with no bacterial growth observed after 24 h. At 4× MIC (8 μg/mL), the complex appeared to be bactericidal at 6 h; however, population regrowth had occurred after 24 h. This behavior was also observed when a second dose of irradiation was applied at the 6 h time point. The bacterial colony which had regrown at 24 h was then collected and cultured to reperform the MIC experiments to check for any compound specific resistance; an identical MIC (2 μg/mL) was obtained for both the nonirradiated and irradiated conditions. Overall, this shows that against *S. aureus* USA300, an MRSA strain, [Ir(**CN01**)_2_(**NN09**)]^+^ appears to have a bactericidal mode of action that is independent of irradiation.

## Conclusions

In this study, we have used a combinatorial and semiautomated approach to rapidly synthesize 78 photoactive iridium(III) complexes and study their antibacterial properties. Previously, these complexes were screened against mammalian cancer cells,^19^ while in this study, we screened them against Gram-positive and Gram-negative bacteria. An initial single-point screen showed that while none of these complexes are active against *E. coli* at concentrations up to 16 μg/mL, a significant proportion (67%) of the compounds showed activity against *S. aureus* MSSA 9144. This very high hit rate indicates that this scaffold is particularly effective at being taken up by Gram-positive bacteria. Interestingly, some of the compounds showed to be active in the dark while others were able to exert their antibacterial activity only after light irradiation. Two rounds of subsequent testing against a panel of five Gram-positive bacteria allowed us to identify two lead compounds with high activity against antibiotic-resistant strains. As discussed in the paper, bacteria find it difficult to develop resistance against photoactive metal complex antibacterials due to their ROS-mediated killing mechanisms.^36^ The time-kill assay showed that, although some population regrowth was observed after 12 h at 4× MIC, there were no changes in the MIC after reculturing and retesting bacteria that survived 24 h exposure, suggesting that no stable mutations were present. These compounds provide a promising lead scaffold that can be optimized to enhance activity against Gram-positive bacteria, widen their spectrum, and improve activity against Gram-negative bacteria through a medicinal chemistry approach. As has been previously reported, compounds with good bacterial uptake (particularly in Gram-negative strains) tend to have protonatable N groups, which is the case for our two lead complexes.^37^ A focused library using the same **CN01** ligand (which largely dictates the photophysical properties of the resulting complexes^19^) and a broader range of **NN** ligands containing different amines, seems a plausible design to generate compounds that are more active (and selective) against bacteria vs mammalian cells.

## Supporting Information Available

Spectroscopic data of the two lead complexes; tables and figures summarizing single-point screen data for *S. aureus* MSSA 9144 with and without irradiation (PDF)
