# Supplementary material for: Discovery of Phototoxic Metal Complexes with Antibacterial Properties via a Combinatorial Approach
Source: Inorg Chem. 2025 Feb 28;64(10):5113–21. doi: 10.1021/acs.inorgchem.4c05414 (PMC11920948; doi:10.1021/acs.inorgchem.4c05414)
Supplement: Supplementary file 1 — ic4c05414_si_001.pdf [file ic4c05414_si_001.pdf]

Discovery of phototoxic metal complexes with antibacterial properties via a combinatorial approach

Timothy Kench,[1] Nasima Chowdhury,[2] Khondaker Miraz Rahman,[2] Ramon Vilar[1]*

[1]Department of Chemistry, Imperial College London, White City Campus, 82 Wood Lane London, W12 OBZ, UK

[2]Institute of Pharmaceutical Science, King's College London, Franklin-Wilkins Building, 150 Stamford Street, London SE1 9NH, UK

Table S1. Single point screen data for *S. aureus MSSA 9144* without irradiation at 16 μg/mL.

Complex	*S. aureus*	DMSO (control)	Categorized absorbance values		
			100% inhibition	>50% inhibition	<50% inhibition
$[Ir(CN01)_2(NN01)]^+$	0.557	0.559	0.012		
$[Ir(CN01)_2(NN02)]^+$	0.529	0.513			0.264
$[Ir(CN01)_2(NN03)]^+$	0.562	0.411	0.063		
$[Ir(CN01)_2(NN04)]^+$	0.568	0.461	0.002		
$[Ir(CN01)_2(NN05)]^+$	0.562	0.446	0.019		
$[Ir(CN01)_2(NN06)]^+$	0.568	0.405			0.314
$[Ir(CN01)_2(NN07)]^+$	0.546	0.416		0.116	
$[Ir(CN01)_2(NN08)]^+$	0.546	0.457		0.179	
$[Ir(CN01)_2(NN09)]^+$	0.562	0.428	-0.006		
$[Ir(CN01)_2(NN11)]^+$	0.562	0.509			0.431
$[Ir(CN01)_2(NN13)]^+$	0.568	0.602	0.001		
$[Ir(CN01)_2(NN14)]^+$	0.562	0.4	-0.016		
$[Ir(CN01)_2(NN15)]^+$	0.568	0.482		0.05	
$[Ir(CN01)_2(NN16)]^+$	0.529	0.462		0.084	
$[Ir(CN01)_2(NN17)]^+$	0.562	0.51	0.004		
$[Ir(CN01)_2(NN18)]^+$	0.562	0.412	0.008		
$[Ir(CN02)_2(NN01)]^+$	0.557	0.467		0.177	
$[Ir(CN02)_2(NN02)]^+$	0.529	0.449			0.879
$[Ir(CN02)_2(NN03)]^+$	0.562	0.431	0.033		
$[Ir(CN02)_2(NN04)]^+$	0.568	0.468	-0.005		
$[Ir(CN02)_2(NN05)]^+$	0.562	0.47	-0.004		
$[Ir(CN02)_2(NN06)]^+$	0.568	0.405		0.077	
$[Ir(CN02)_2(NN07)]^+$	0.546	0.415	0.061		
$[Ir(CN02)_2(NN08)]^+$	0.546	0.619			0.321
$[Ir(CN02)_2(NN09)]^+$	0.562	0.434	-0.013		
$[Ir(CN02)_2(NN11)]^+$	0.562	0.399	-0.005		
$[Ir(CN03)_2(NN01)]^+$	0.557	0.412			0.425
$[Ir(CN03)_2(NN02)]^+$	0.529	0.462			0.442
$[Ir(CN03)_2(NN03)]^+$	0.562	0.435			0.263
$[Ir(CN03)_2(NN04)]^+$	0.568	0.482	-0.002		
$[Ir(CN03)_2(NN05)]^+$	0.562	0.438			0.396
$[Ir(CN03)_2(NN06)]^+$	0.568	0.408			0.2
$[Ir(CN03)_2(NN07)]^+$	0.546	0.437			0.399
$[Ir(CN03)_2(NN08)]^+$	0.546	0.426			0.394
$[Ir(CN03)_2(NN09)]^+$	0.562	0.406			0.216
$[Ir(CN03)_2(NN11)]^+$	0.562	0.558			0.374
$[Ir(CN04)_2(NN01)]^+$	0.557	0.452			0.429
$[Ir(CN04)_2(NN02)]^+$	0.529	0.513			0.421

$[Ir(CN04)_2(NN03)]^+$	0.562	0.581			0.565
$[Ir(CN04)_2(NN04)]^+$	0.568	0.47			0.511
$[Ir(CN04)_2(NN05)]^+$	0.562	0.433		0.146	
$[Ir(CN04)_2(NN06)]^+$	0.568	0.483			0.609
$[Ir(CN04)_2(NN07)]^+$	0.546	0.477			0.499
$[Ir(CN04)_2(NN08)]^+$	0.546	0.462			0.413
$[Ir(CN04)_2(NN09)]^+$	0.562	0.451			0.494
$[Ir(CN04)_2(NN11)]^+$	0.562	0.435			0.268
$[Ir(CN05)_2(NN01)]^+$	0.557	0.449			0.44
$[Ir(CN05)_2(NN02)]^+$	0.529	0.405			0.343
$[Ir(CN05)_2(NN03)]^+$	0.562	0.419			0.286
$[Ir(CN05)_2(NN04)]^+$	0.568	0.465	-0.005		
$[Ir(CN05)_2(NN05)]^+$	0.562	0.437		0.191	
$[Ir(CN05)_2(NN06)]^+$	0.568	0.664			0.374
$[Ir(CN05)_2(NN07)]^+$	0.546	0.391			0.416
$[Ir(CN05)_2(NN08)]^+$	0.546	0.447		0.109	
$[Ir(CN05)_2(NN09)]^+$	0.562	0.44			0.466
$[Ir(CN05)_2(NN11)]^+$	0.562	0.449			0.135
$[Ir(CN06)_2(NN01)]^+$	0.557	0.418			0.407
$[Ir(CN06)_2(NN02)]^+$	0.529	0.408			0.419
$[Ir(CN06)_2(NN03)]^+$	0.562	0.673			0.521
$[Ir(CN06)_2(NN04)]^+$	0.568	0.431			0.213
$[Ir(CN06)_2(NN05)]^+$	0.562	0.468			0.372
$[Ir(CN06)_2(NN06)]^+$	0.568	0.523			0.517
$[Ir(CN06)_2(NN07)]^+$	0.546	0.44			0.366
$[Ir(CN06)_2(NN08)]^+$	0.546	0.412			0.463
$[Ir(CN06)_2(NN09)]^+$	0.562	0.439			0.406
$[Ir(CN06)_2(NN11)]^+$	0.562	0.424			0.518
$[Ir(CN06)_2(NN13)]^+$	0.546	0.402	-0.005		
$[Ir(CN06)_2(NN14)]^+$	0.546	0.395			0.482
$[Ir(CN06)_2(NN15)]^+$	0.557	0.422			0.4
$[Ir(CN06)_2(NN16)]^+$	0.562	0.668			0.577
$[Ir(CN06)_2(NN17)]^+$	0.546	0.428			0.398
$[Ir(CN06)_2(NN18)]^+$	0.546	0.4		0.105	
$[Ir(CN07)_2(NN13)]^+$	0.568	0.473	-0.002		
$[Ir(CN07)_2(NN14)]^+$	0.562	0.402			0.455
$[Ir(CN07)_2(NN15)]^+$	0.568	0.484	0.001		
$[Ir(CN07)_2(NN16)]^+$	0.529	0.449			0.447
$[Ir(CN07)_2(NN17)]^+$	0.562	0.476			0.279
$[Ir(CN07)_2(NN18)]^+$	0.562	0.458	-0.011		

Table S2. Single point screen data for *S. aureus MSSA 9144* after irradiation at 16 μg/mL.

Complex	*S. aureus*	DMSO (control)	Categorized absorbance values		
			100% inhibition	>50% inhibition	<50% inhibition
$[Ir(CN01)_2(NN01)]^+$	0.495	0.442	0.08		
$[Ir(CN01)_2(NN02)]^+$	0.542	0.528			0.273
$[Ir(CN01)_2(NN03)]^+$	0.406	0.445	0.002		
$[Ir(CN01)_2(NN04)]^+$	0.416	0.372			0.188
$[Ir(CN01)_2(NN05)]^+$	0.471	0.41	-0.003		
$[Ir(CN01)_2(NN06)]^+$	0.423	0.402			0.256
$[Ir(CN01)_2(NN07)]^+$	0.474	0.404	0.071		
$[Ir(CN01)_2(NN08)]^+$	0.445	0.403		0.151	
$[Ir(CN01)_2(NN09)]^+$	0.395	0.37	0.026		
$[Ir(CN01)_2(NN11)]^+$	0.402	0.404	-0.001		
$[Ir(CN01)_2(NN13)]^+$	0.542	0.453	0.003		
$[Ir(CN01)_2(NN14)]^+$	0.416	0.468	0		
$[Ir(CN01)_2(NN15)]^+$	0.42	0.393	-0.006		
$[Ir(CN01)_2(NN16)]^+$	0.426	0.388			0.368
$[Ir(CN01)_2(NN17)]^+$	0.474	0.388	0.004		
$[Ir(CN01)_2(NN18)]^+$	0.423	0.378	-0.009		
$[Ir(CN02)_2(NN01)]^+$	0.445	0.413	-0.016		
$[Ir(CN02)_2(NN02)]^+$	0.395	0.398			0.216
$[Ir(CN02)_2(NN03)]^+$	0.41	0.409	-0.007		
$[Ir(CN02)_2(NN04)]^+$	0.406	0.426	-0.003		
$[Ir(CN02)_2(NN05)]^+$	0.447	0.407	-0.003		
$[Ir(CN02)_2(NN06)]^+$	0.402	0.444	-0.007		
$[Ir(CN02)_2(NN07)]^+$	0.485	0.413	0.062		
$[Ir(CN02)_2(NN08)]^+$	0.611	0.46		0.119	
$[Ir(CN02)_2(NN09)]^+$	0.414	0.399	-0.006		
$[Ir(CN02)_2(NN11)]^+$	0.411	0.426	0.002		
$[Ir(CN03)_2(NN01)]^+$	0.474	0.391	-0.011		
$[Ir(CN03)_2(NN02)]^+$	0.411	0.42			0.404
$[Ir(CN03)_2(NN03)]^+$	0.423	0.422			0.481
$[Ir(CN03)_2(NN04)]^+$	0.426	0.404	-0.002		
$[Ir(CN03)_2(NN05)]^+$	0.474	0.397	0.001		
$[Ir(CN03)_2(NN06)]^+$	0.41	0.387			0.314
$[Ir(CN03)_2(NN07)]^+$	0.447	0.401	0.043		
$[Ir(CN03)_2(NN08)]^+$	0.442	0.419	-0.02		
$[Ir(CN03)_2(NN09)]^+$	0.42	0.376		0.147	
$[Ir(CN03)_2(NN11)]^+$	0.542	0.45	0		
$[Ir(CN04)_2(NN01)]^+$	0.447	0.405			0.399
$[Ir(CN04)_2(NN02)]^+$	0.416	0.408			0.448

$[Ir(CN04)_2(NN03)]^+$	0.495	0.467			0.507
$[Ir(CN04)_2(NN04)]^+$	0.402	0.4			0.453
$[Ir(CN04)_2(NN05)]^+$	0.442	0.396	0.003		
$[Ir(CN04)_2(NN06)]^+$	0.395	0.397			0.475
$[Ir(CN04)_2(NN07)]^+$	0.611	0.501			0.454
$[Ir(CN04)_2(NN08)]^+$	0.445	0.376		0.18	
$[Ir(CN04)_2(NN09)]^+$	0.406	0.42			0.424
$[Ir(CN04)_2(NN11)]^+$	0.414	0.445		0.324	0.324
$[Ir(CN05)_2(NN01)]^+$	0.471	0.398			0.331
$[Ir(CN05)_2(NN02)]^+$	0.42	0.386			0.416
$[Ir(CN05)_2(NN03)]^+$	0.402	0.421			0.227
$[Ir(CN05)_2(NN04)]^+$	0.423	0.434		0.161	
$[Ir(CN05)_2(NN05)]^+$	0.485	0.402	-0.005		
$[Ir(CN05)_2(NN06)]^+$	0.542	0.474			0.301
$[Ir(CN05)_2(NN07)]^+$	0.445	0.395		0.121	
$[Ir(CN05)_2(NN08)]^+$	0.495	0.397		0.183	
$[Ir(CN05)_2(NN09)]^+$	0.416	0.427	-0.003		
$[Ir(CN05)_2(NN11)]^+$	0.395	0.406	0.004		
$[Ir(CN06)_2(NN01)]^+$	0.474	0.391	0.082		
$[Ir(CN06)_2(NN02)]^+$	0.414	0.415			0.226
$[Ir(CN06)_2(NN03)]^+$	0.611	0.55	0.004		
$[Ir(CN06)_2(NN04)]^+$	0.41	0.421	0.003		
$[Ir(CN06)_2(NN05)]^+$	0.445	0.446	-0.003		
$[Ir(CN06)_2(NN06)]^+$	0.411	0.434	-0.001		
$[Ir(CN06)_2(NN07)]^+$	0.442	0.375		0.159	
$[Ir(CN06)_2(NN08)]^+$	0.474	0.366			0.225
$[Ir(CN06)_2(NN09)]^+$	0.426	0.437		0.141	
$[Ir(CN06)_2(NN11)]^+$	0.42	0.437	-0.011		
$[Ir(CN06)_2(NN13)]^+$	0.471	0.392	-0.007		
$[Ir(CN06)_2(NN14)]^+$	0.474	0.425	0.004		
$[Ir(CN06)_2(NN15)]^+$	0.485	0.429	-0.014		
$[Ir(CN06)_2(NN16)]^+$	0.611	0.487			0.307
$[Ir(CN06)_2(NN17)]^+$	0.445	0.43			0.244
$[Ir(CN06)_2(NN18)]^+$	0.495	0.407	-0.001		
$[Ir(CN07)_2(NN13)]^+$	0.411	0.365	-0.004		
$[Ir(CN07)_2(NN14)]^+$	0.426	0.436	0		
$[Ir(CN07)_2(NN15)]^+$	0.414	0.396	-0.004		
$[Ir(CN07)_2(NN16)]^+$	0.406	0.428			0.483
$[Ir(CN07)_2(NN17)]^+$	0.445	0.423	-0.001		
$[Ir(CN07)_2(NN18)]^+$	0.41	0.393	-0.006		

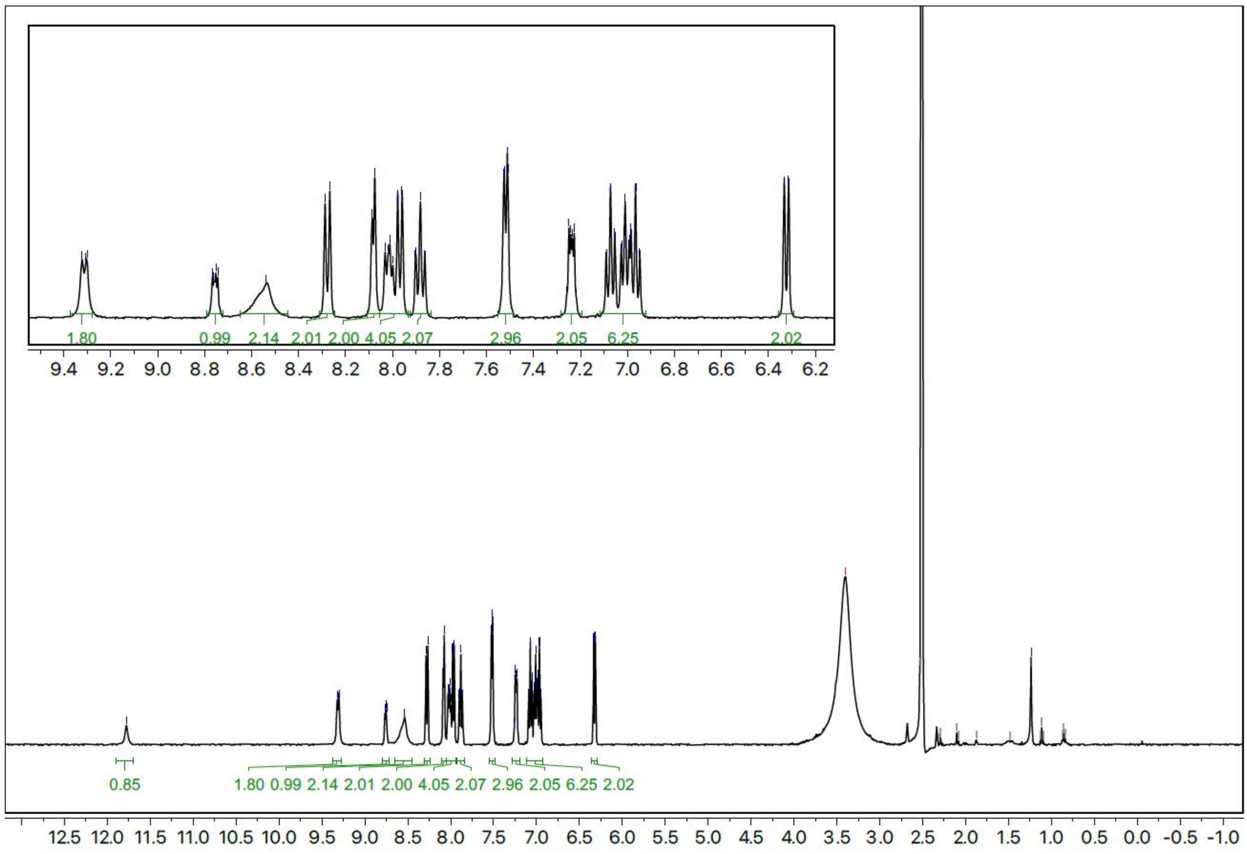

Figure S1. ^{1}H NMR of [Ir(**CN01**)$_2$(**NN09**)]$^+$ in DMSO-d$_6$.

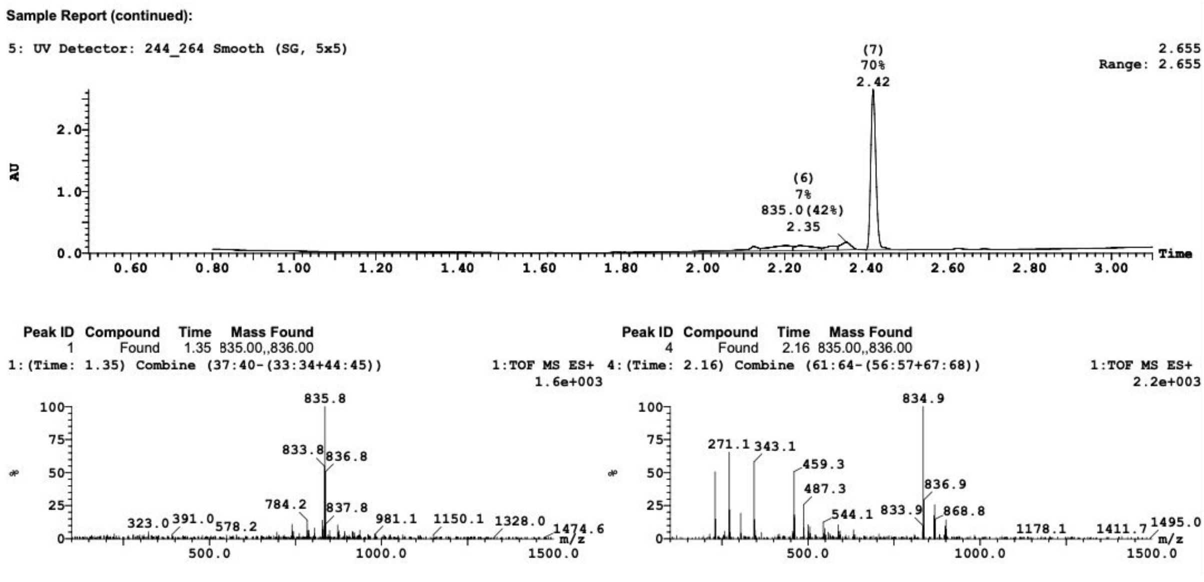

Figure S2. LCMS of [Ir(**CN01**)$_2$(**NN09**)]$^+$, 5-95% MeCN in H_2O with 0.1% formic acid.

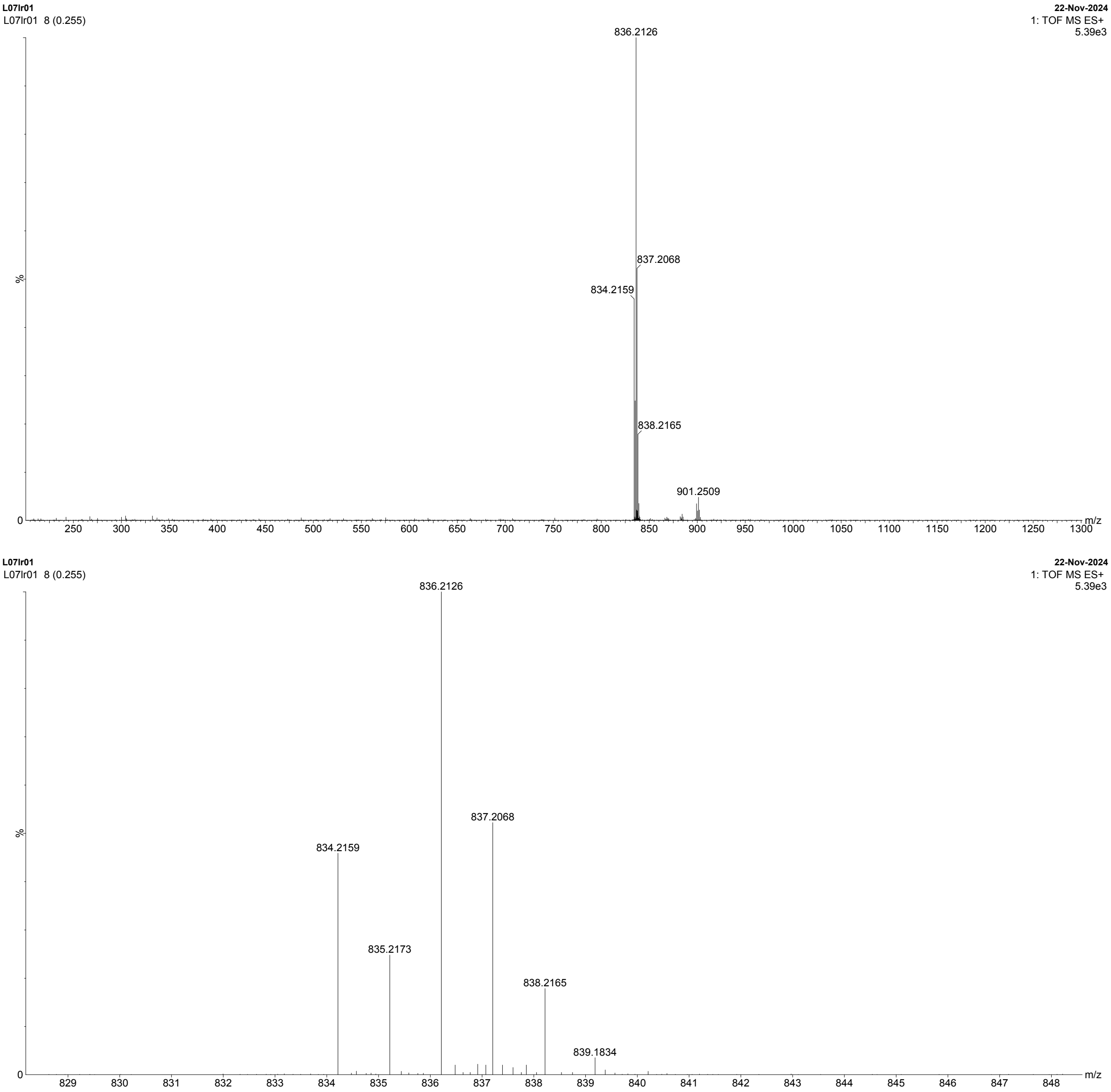

Figure S3. High Resolution ESI-MS of [Ir(**CN01**)$_2$(**NN09**)]$^+$.

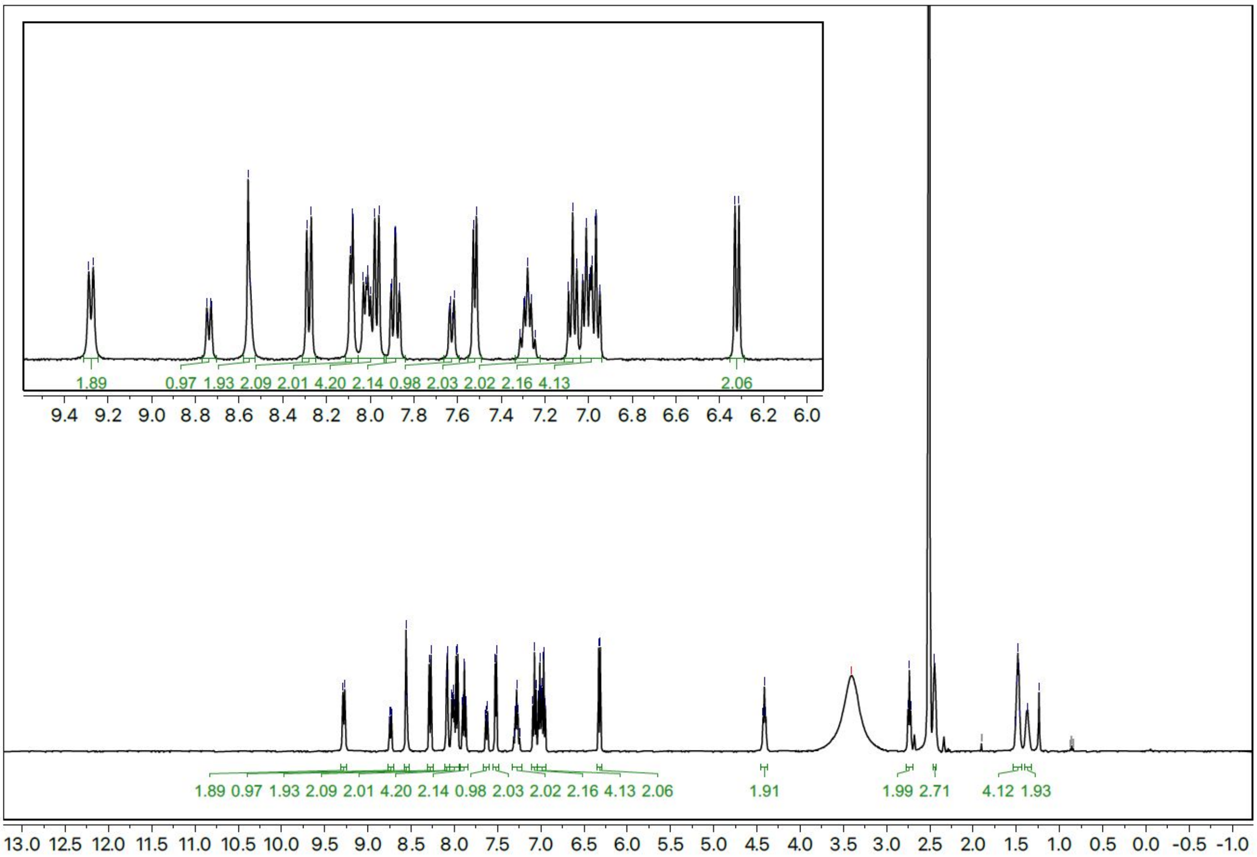

Figure S4. ^{1}H NMR of $[Ir(\mathbf{CN01})_2(\mathbf{NN18})]^+$ in DMSO-d_6.

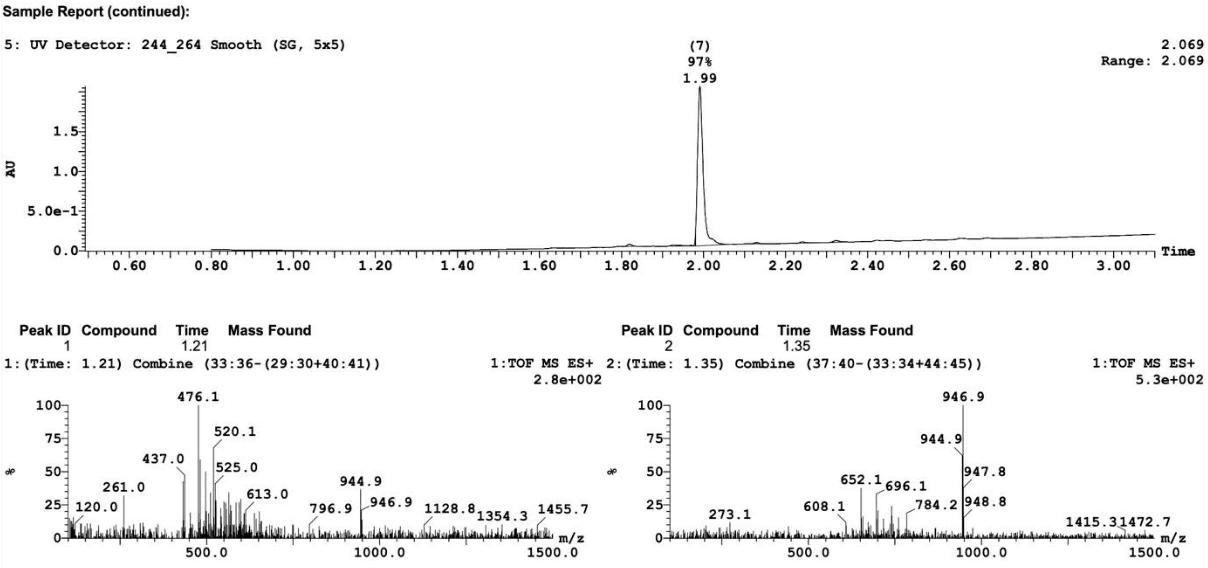

Figure S5. LCMS of $[Ir(\mathbf{CN01})_2(\mathbf{NN18})]^+$, 5-95% MeCN in H_2O with 0.1% formic acid.

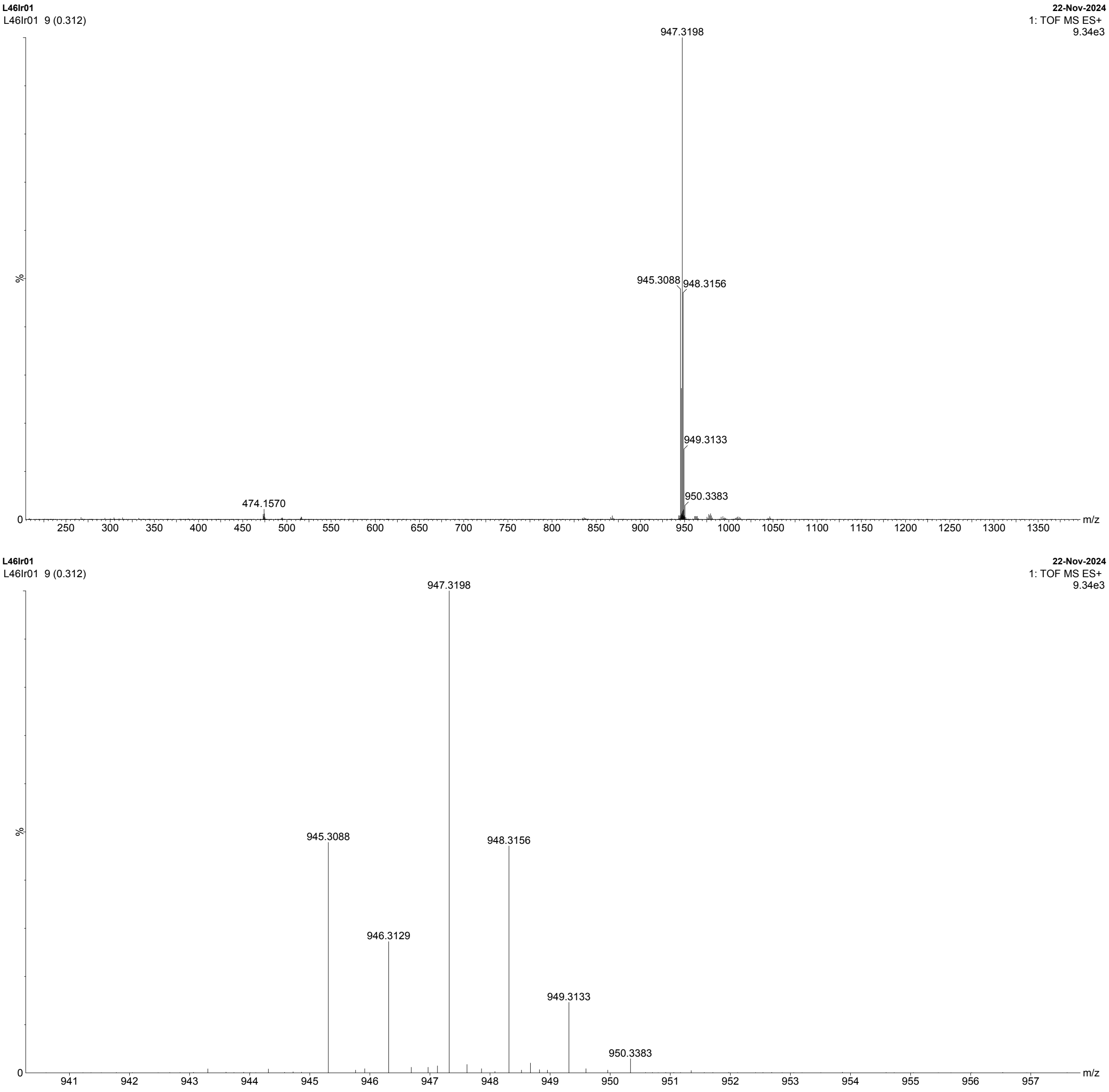

Figure S6. High Resolution ESI-MS of [Ir(**CN01**)$_2$(**NN18**)]$^+$.

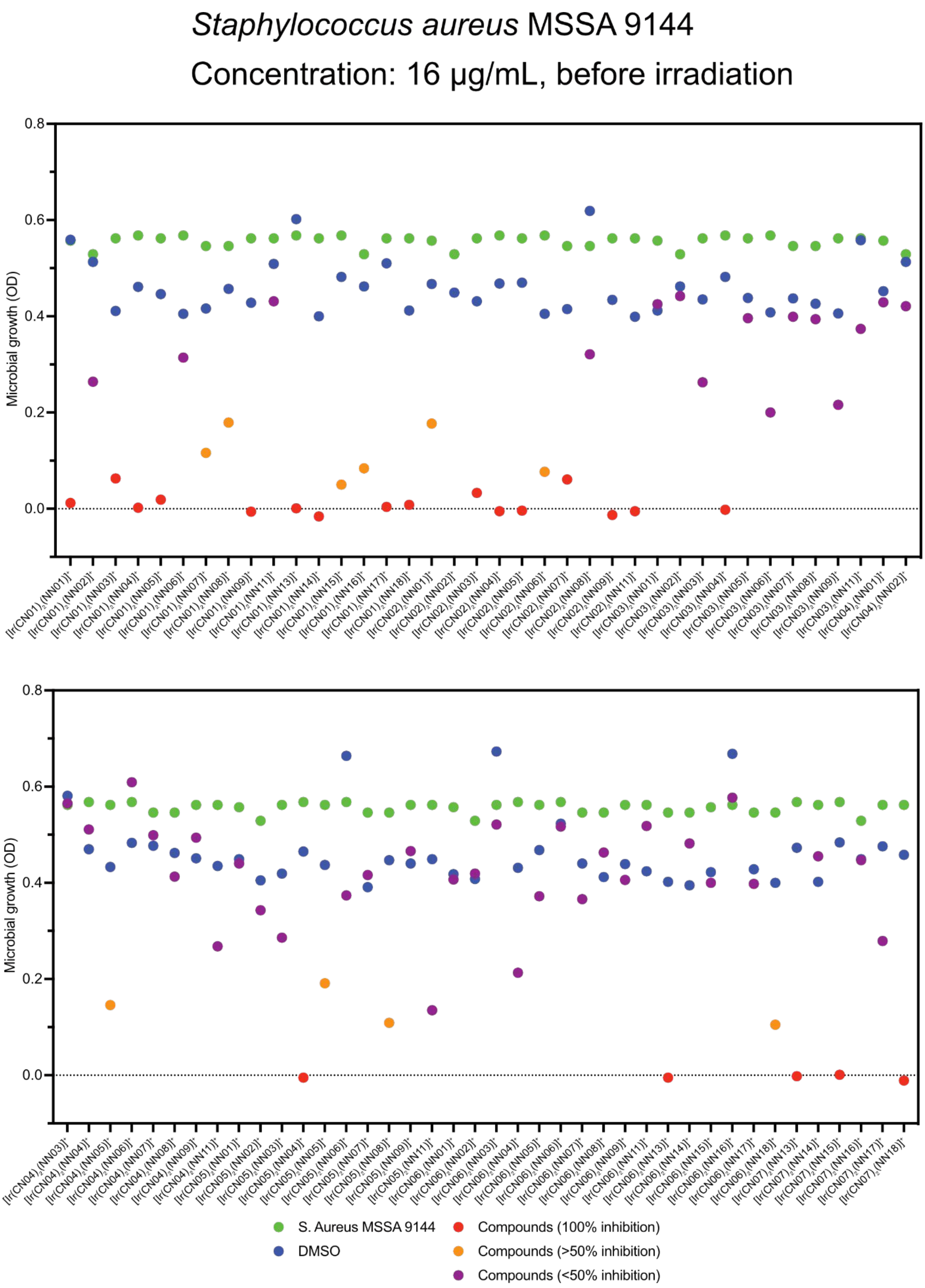

Figure S7. Effect of the compounds and DMSO on bacterial growth without irradiation.

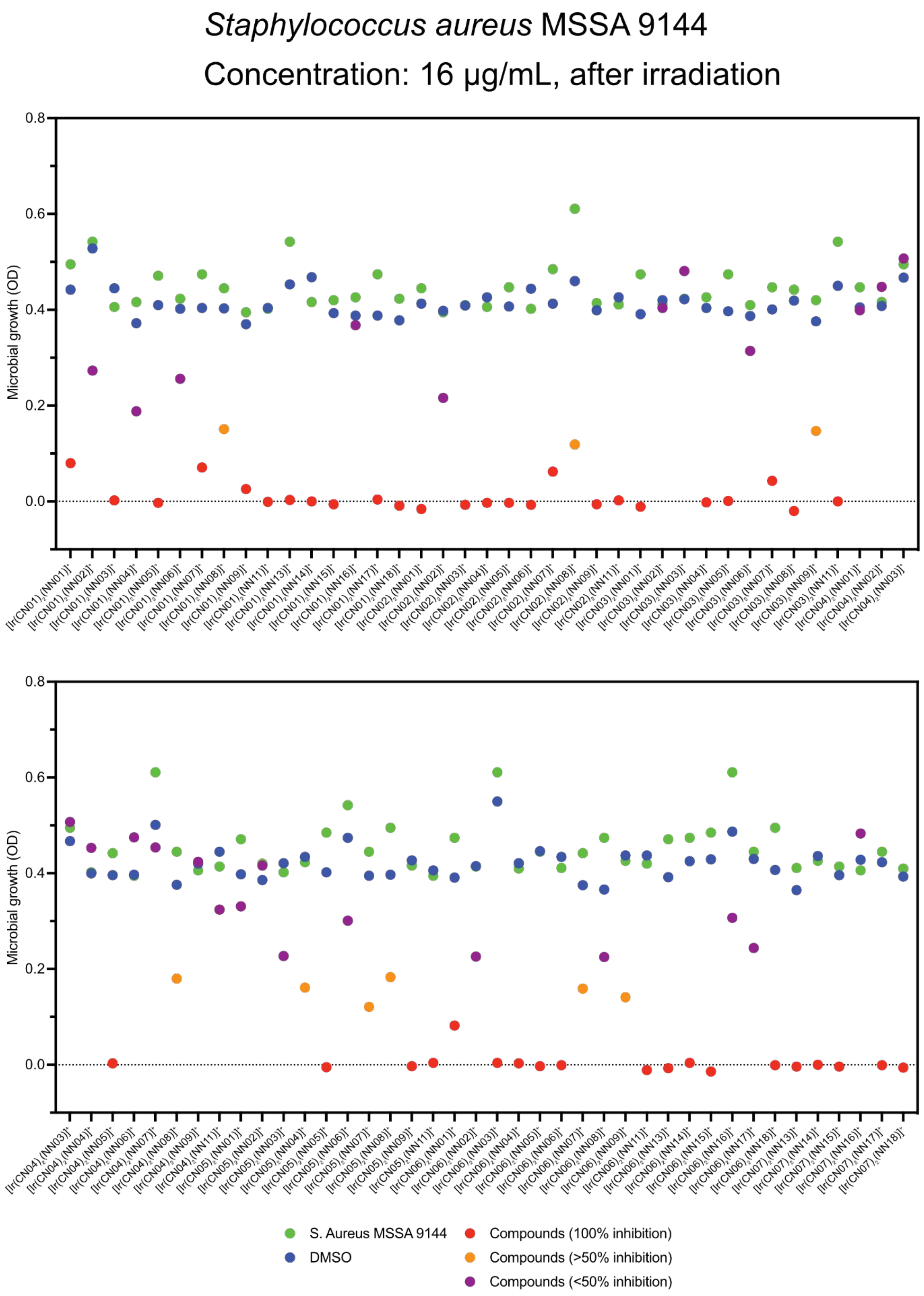

Figure S8. Effect of the compounds and DMSO on bacterial growth without irradiation.
